# Human RecQ Helicases in DNA Double-Strand Break Repair

**DOI:** 10.3389/fcell.2021.640755

**Published:** 2021-02-25

**Authors:** Huiming Lu, Anthony J. Davis

**Affiliations:** Division of Molecular Radiation Biology, Department of Radiation Oncology, UT Southwestern Medical Center, Dallas, TX, United States

**Keywords:** RecQ helicase, RECQL1, BLM, WRN, RECQL4, RECQL5, DNA double-strand break repair, genome stability

## Abstract

RecQ DNA helicases are a conserved protein family found in bacteria, fungus, plants, and animals. These helicases play important roles in multiple cellular functions, including DNA replication, transcription, DNA repair, and telomere maintenance. Humans have five RecQ helicases: RECQL1, Bloom syndrome protein (BLM), Werner syndrome helicase (WRN), RECQL4, and RECQL5. Defects in BLM and WRN cause autosomal disorders: Bloom syndrome (BS) and Werner syndrome (WS), respectively. Mutations in RECQL4 are associated with three genetic disorders, Rothmund–Thomson syndrome (RTS), Baller–Gerold syndrome (BGS), and RAPADILINO syndrome. Although no genetic disorders have been reported due to loss of RECQL1 or RECQL5, dysfunction of either gene is associated with tumorigenesis. Multiple genetically independent pathways have evolved that mediate the repair of DNA double-strand break (DSB), and RecQ helicases play pivotal roles in each of them. The importance of DSB repair is supported by the observations that defective DSB repair can cause chromosomal aberrations, genomic instability, senescence, or cell death, which ultimately can lead to premature aging, neurodegeneration, or tumorigenesis. In this review, we will introduce the human RecQ helicase family, describe in detail their roles in DSB repair, and provide relevance between the dysfunction of RecQ helicases and human diseases.

## DNA Double-Strand Breaks and Repair Pathways

**FIGURE 1:**
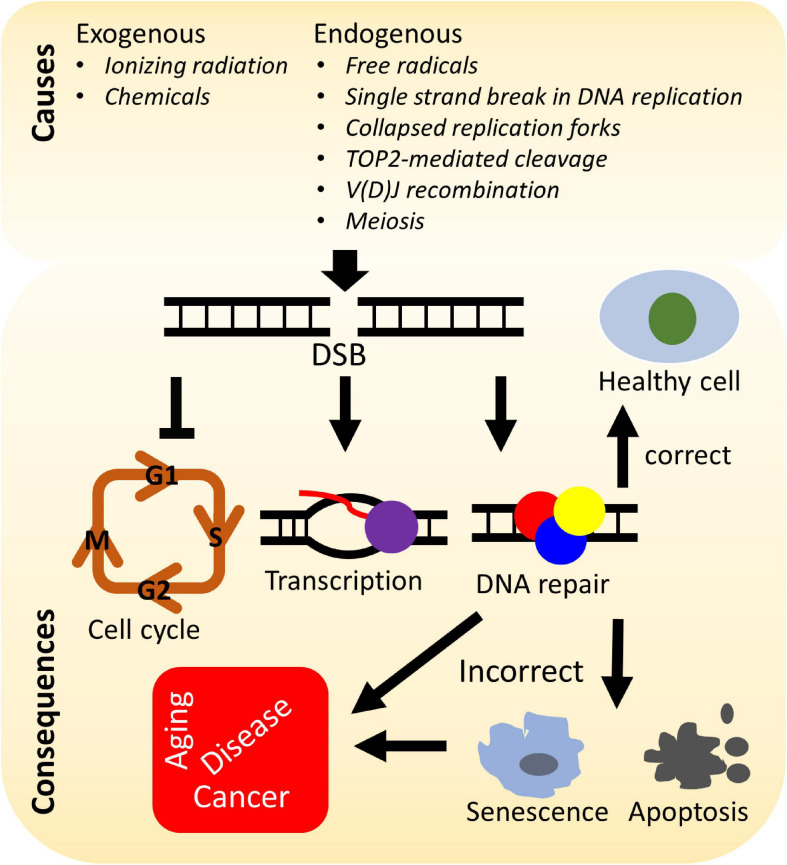
Causes and consequences of DNA double-strand breaks (DSBs). DSBs arise from various stresses by endogenous or exogenous factors and can lead to arrest of the cell cycle, transcription, activation of the DNA damage response, and repair of the DNA damage. Incorrectly repaired or unrepaired DSBs can result in cellular senescence, apoptosis, premature aging, genetic disorders, and/or tumorigenesis.

**FIGURE 2:**
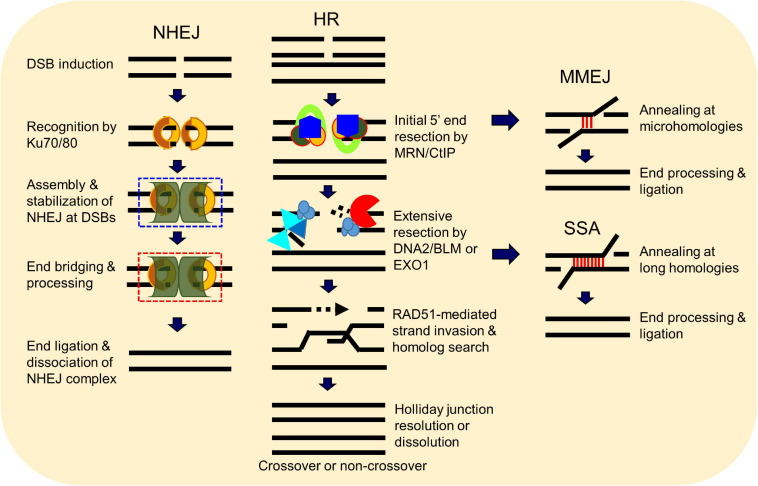
Double-strand break (DSB) repair pathways. DSBs in mammalian cells are able to be repaired by at least four pathways, including non-homologous end joining (NHEJ), homologous recombination (HR), microhomology-mediated end joining (MMEJ), and single-strand annealing (SSA). HR and NHEJ are the dominant DSB repair pathways in normal cells, but the two minor pathways SSA and MMEJ can occur under certain circumstances. Choice between these repair pathways is tightly regulated.

A number of elegant mechanisms have evolved that repair the vast number of DNA lesions an organism encounters each day. DNA repair mechanisms are described as guardians of the human genome because DNA is the template for the fundamental processes of replication and transcription, and preserving the integrity of genomic DNA ensures faithful propagation of genetic material and transmission to daughter cells (Hoeijmakers, 2009; Ciccia and Elledge, 2010; Tubbs and Nussenzweig, 2017). Arguably, the most important DNA repair mechanisms are those that repair DNA double-strand breaks (DSBs) (Ciccia and Elledge, 2010; Ceccaldi et al., 2016; Scully et al., 2019). DSBs are generated during endogenous events such as after the collapse of replication forks (Bouwman and Crosetto, 2018), SPO11-induced DSB formation during meiosis (Tock and Henderson, 2018), V(D)J (variable, diversity, and joining) recombination (Chi et al., 2020), and *via* reactive oxygen species generated during metabolism, as well as from various exogenous stresses which include ionizing radiation (IR) and cancer chemotherapeutic agents (Tubbs and Nussenzweig, 2017) (Figure 1). Unrepaired or misrepaired DSBs can cause chromosomal aberrations, genomic instability, senescence, or cell death, further leading to premature aging, neurodegeneration, or tumorigenesis (Figure 1) (White and Vijg, 2016; Tubbs and Nussenzweig, 2017; Taylor et al., 2019). To overcome severe consequences from DSBs, mammalian cells have evolved at least four pathways to repair this type of DNA lesion, termed non-homologous end joining (NHEJ), homologous recombination (HR), and the alternative end-joining pathways, microhomology-mediated end joining (MMEJ) and single-strand annealing (SSA) (Figure 2). In the following sections, we will give a brief overview of each DSB repair pathway.

### Non-homologous End Joining

Non-homologous end joining (NHEJ) is the major pathway responsible for the repair of two-ended DSBs generated by IR, restriction enzymes, and those intentionally generated for V(D)J and class switch recombination during T and B cell lymphocyte development (Davis and Chen, 2013; Davis et al., 2014; Scully et al., 2019). NHEJ is a flexible process that directs re-ligation of the broken DNA molecule in a template-independent manner and is active in all phases of the cell cycle (Davis et al., 2014; Pannunzio et al., 2018). Initiation of NHEJ occurs when the ring-shaped Ku heterodimer, composed of the Ku70 and Ku80 proteins, recognizes and binds to the DSB in a sequence-independent manner (Fell and Schild-Poulter, 2015). Once bound to the DSB ends, Ku then functions as a scaffold to recruit the NHEJ machinery to the damage site. In particular, Ku70/80 directly recruits DNA-dependent protein kinase catalytic subunit (DNA-PK_cs_) to the DNA ends to form the DNA-PK complex, resulting in the activation of DNA-PK_cs_ kinase activity (Davis et al., 2014). DNA-PK_cs_ promotes NHEJ and phosphorylates the histone H2AX and the chromatin remodeler KAP1 to promote chromatin relaxation proximal to the DSB to facilitate recruitment of the DNA damage response and repair machinery to the DNA damage site (Lu et al., 2019). If the ends of the DSB are not compatible for ligation, different enzymes are required, including those that resect DNA ends, fill in gaps, or remove blocking end groups, to process the DNA ends to allow ligation (Pannunzio et al., 2018). The terminal step in NHEJ is the ligation of the broken DNA ends by the DNA ligase IV(LIG4)/X-ray cross-complementing protein 4 (XRCC4) complex with the assistance of the XRCC4-like factor (XLF) (Ellenberger and Tomkinson, 2008). NHEJ is not intrinsically inaccurate, but the quality of end joining is dictated by the structure of the DSB ends as small insertions or deletions can be generated if end processing by nucleases and polymerases is required (Davis and Chen, 2013; Pannunzio et al., 2018; Scully et al., 2019).

### Homologous Recombination

Homologous recombination (HR) requires a homologous DNA sequence to serve as a template for DNA synthesis-dependent repair. It is an accurate process as it employs DNA sequences homologous to/near the broken ends to drive repair, predominantly using the sister chromatid as a template for DSB repair rather than the homologous chromosome. As a sister chromatid is available after DNA replication, HR predominantly occurs in mid-S to the early G2 phase of the cell cycle (Ceccaldi et al., 2016; Hustedt and Durocher, 2016). HR is initiated by the MRE11/RAD50/NBS1 (MRN) complex in conjunction with CtIP *via* the endonuclease and 3′–5′ exonuclease activities of MRE11 (Lengsfeld et al., 2007; Sartori et al., 2007; Garcia et al., 2011; Cannavo and Cejka, 2014; Deshpande et al., 2020). The endonuclease activity of MRE11 generates a nick in the double-stranded DNA (dsDNA) near the DSB site, followed by its 3′–5′ exonuclease activity generating a short section of single-stranded DNA (ssDNA) next to the nick. This is followed by extensive resection by exonuclease 1 (EXO1) and/or the nuclease DNA2 with the RECQ helicase Bloom syndrome protein (BLM) to produce a long 3′ overhang (Mimitou and Symington, 2008; Nimonkar et al., 2011; Symington, 2014; Ronato et al., 2020). The resulting 3′ ssDNA is rapidly coated by the ssDNA-binding protein replication protein A (RPA), which is subsequently replaced by RAD51 *via* assistance by BRCA2 and DSS1 (Gudmundsdottir et al., 2004; Yang et al., 2005; Jensen et al., 2010; Thorslund et al., 2010; Zhao et al., 2015). RAD51 binds to ssDNA, forming a helical RAD51–ssDNA nucleoprotein filament that is capable of homology search and invasion of a homologous DNA sequence. If sufficient base pairing occurs between the invading RAD51-coated strand and the invaded DNA molecule, the non-base-paired strand of the invaded molecule is displaced in the form of a displacement loop (D-loop). A DNA polymerase extends the 3′-end of the invasion strand past the break using the invaded homologous strand as a template, followed by resolution of the Holliday junction from the extended D-loop by resolvases and dissolution by BLM-TOP3a-RMI1/2 complex, annealing, and ligation of the extended invasion strand to the other end of the DSB on the original DNA molecule (Wright et al., 2018; Scully et al., 2019).

### Alternative End-Joining Pathways

Non-homologous end joining and HR are the dominant DSB repair pathways, but there are two minor alternative end joining pathways called microhomology-mediated end joining (MMEJ) and single-strand annealing (SSA). MMEJ and SSA were initially identified in cells that were deficient in NHEJ and/or HR, and thus, both were believed to be strictly “backup pathways” (Fairman et al., 1992; Boulton and Jackson, 1996; Mason et al., 1996; Kabotyanski et al., 1998), but it has been found that, even in NHEJ- and HR-proficient cells, a small percentage of DSBs are repaired by these alternative pathways (Truong et al., 2013). Similar to NHEJ, the MMEJ and SSA pathways terminate with direct ligation of the two ends of the broken DNA, but MMEJ and SSA are distinct from NHEJ as they function completely independently of the Ku heterodimer and DNA-PK_*cs*_, require components of the HR machinery, and require longer tracts of microhomology to mediate repair (Sallmyr and Tomkinson, 2018). The factors and processes required for MMEJ and SSA are not well defined, but both initiate with the binding of PARP1 to the DSB ends and each requires DNA end resection (Bennardo et al., 2008; Xie et al., 2009; Lee-Theilen et al., 2011; Zhang and Jasin, 2011; Truong et al., 2013). Similar to HR, DNA end resection is initiated by the MRN–CtIP complex in MMEJ and SSA, but the enzymes required for long DNA end resection are not clearly defined, with speculation being that EXO1 and/or DNA2/BLM mediate this process (Sallmyr and Tomkinson, 2018). Each alternative end-joining pathway requires differing amounts of sequence homology to align the DNA. MMEJ requires a short region of complimentary sequence, 2–20 nucleotides, called microhomology sequences, whereas SSA needs >25 nucleotides of homologous sequence, which typically reside within tandem repeats (Bhargava et al., 2016). During SSA, the generated 3′ ssDNAs are annealed by RAD52 *via* alignment of homologous sequences, whereas multiple enzymes are responsive for end bridging and annealing in MMEJ, including the MRN complex, PARP1, and Polθ (Sallmyr and Tomkinson, 2018). Once aligned, the non-complementary sequences generate 3′ ssDNA overhangs that are removed by nucleases. Both MMEJ and SSA complete with gap filling and DNA ligation by DNA polymerases and DNA ligases, but the exact enzymes and mechanisms that drive these processes are not well defined (Sallmyr and Tomkinson, 2018; Patterson-Fortin and D’Andrea, 2020). The repair of DSBs by MMEJ and SSA are intrinsically mutagenic as they cause deletions and rearrangements, resulting in genomic instability (Sallmyr and Tomkinson, 2018; Patterson-Fortin and D’Andrea, 2020).

## Human RecQ Helicases

**FIGURE 3:**
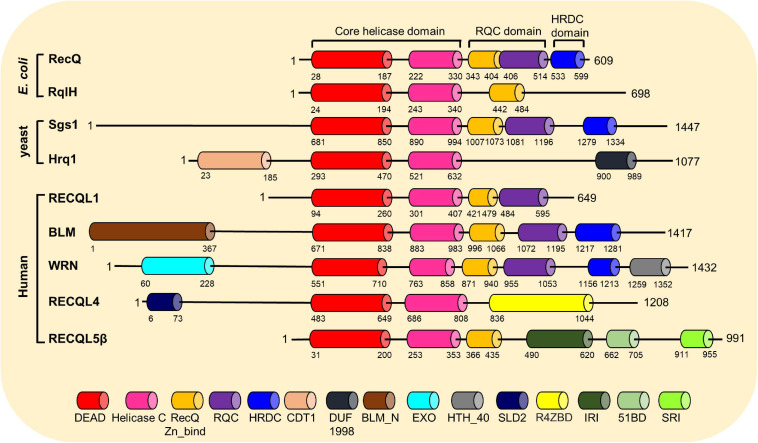
Conserved motifs and domains of RecQ helicases from *Escherichia coli*, budding yeast, and humans. The RecQ helicases’ motifs and domains are presented mainly based on the annotations by the GenomeNet Database (https://www.genome.jp/), with information from published literatures. *DEAD*, DEAD/DEAH box helicase; *Helicase C*, helicase conserved C-terminal motif; *RecQ-Zn-Bind*, RecQ Zn^2+^-binding motif; *RQC*, RecQ-C-terminal domain; *HRDC*, helicase and RNase D-like C-terminal domain; *CDT1*, DNA replication factor CDT1 like; *DUF1998*, domain of unknown function (DUF1998); *BLM_N*, N-terminal region of Bloom syndrome protein; *EXO*, DNA_POLA_EXO1, 3′–5′ exonuclease domain; *HTH_40*, helix-turn-helix domain; *SLD2*, DNA replication and checkpoint protein; *R4ZBD*, RECQL4-Zn^2+^-binding motif; *IRI*, internal RNAPII-interacting domain; *51BD*, RAD51-binding domain; *SRI*, SET2-RPB1 interaction motif.

Helicases are a ubiquitous family of molecular motors that unwind DNA, RNA, and DNA–RNA duplexes. They play essential roles in DNA and RNA metabolism, including DNA replication, transcription, DNA repair, translation, RNA maturation, ribosome synthesis, and splicing. A conserved helicase family is the RecQ family, which is named after the prototypical member found in *Escherichia coli* called RecQ (Cobb and Bjergbaek, 2006; Croteau et al., 2014). RecQ helicases possess 3′ to 5′ directionality and can unwind a variety of DNA structures including B-form DNA, forked DNA duplexes, D-loops, DNA junctions, and G-quadruplexes (Croteau et al., 2014). Furthermore, they promote the annealing of complementary ssDNAs and branch migration of Holliday junctions. Each RecQ helicase shares the highly conserved core helicase domain (DEAD/DEAH box, helicase conserved C-terminal domain), with the majority of the family members containing the RecQ C-terminal (RQC) domain, and the helicase and RNase D-like C-terminal (HRDC) domain is shared among members (Figure 3). An important function of RecQ helicases is that they are essential to maintain genome stability as they act at the interface between DNA replication, DNA recombination, DNA repair, telomere maintenance, and transcription (Bohr, 2008; Croteau et al., 2014; Urban et al., 2017). Besides RecQ, *E. coli* has another RecQ-like helicase, named RqlH, which does not appear in the K12 strain of *E. coli* but in many other strains (Russell and Mulvey, 2015). Two RecQ helicases, Sgs1 and Hrq1, have been identified in the budding yeast *Saccharomyces cerevisiae* as homologs to human BLM and RECQL4, respectively (Watt et al., 1996; Choi et al., 2013; Bochman et al., 2014; Rogers et al., 2017). Human cells have five distinct RecQ helicases, named RECQL1, BLM, Werner syndrome helicase (WRN), RECQL4, and RECQL5. All of them have the conserved helicase core domain, but only BLM and WRN possess the HRDC domain, and RECQL1, BLM, and WRN have the typical RQC domain (Figure 3). Notably, the human RecQ helicases play important functions in nearly all DNA repair pathways, in particular those required for the repair of DSBs (Hickson, 2003; Bohr, 2008; Croteau et al., 2014). The role of human RecQ helicases in DSB repair is supported by the observation that defects in these enzymes result in a number of distinct human genetic disorders, premature aging, and/or carcinogenesis, which may be driven due to defective DSB repair (Datta et al., 2020; Oshima et al., 2018). These RecQ helicases participate in multiple DSB repair pathways by physically and functionally interacting with key players in these pathways (Table 1). In this review, we will focus on the important roles of human RecQ helicases in DSB repair and maintenance of genome stability, as well as the biological relevance between these cellular functions and the mechanisms underlying RecQ-associated disorders and cancers.

**TABLE 1:** RecQ helicase-interacting proteins in DSB repair pathways.

	Pathway	Interacting proteins	Function	References
RECQL1	NHEJ	Ku70, Ku80, SHDL2	Promotes NHEJ by interacting with Ku70/80	Parvathaneni et al., 2013; Howard et al., 2015; Findlay et al., 2018
	HR	RAD51	Undefined function in HR	Sharma and Brosh Jr., 2007
	MMEJ	PARP1	Promotes MMEJ by an unknown mechanism	Sharma et al., 2012; Berti et al., 2013; Howard et al., 2015
BLM	HR	DNA2, EXO1, RPA, TOP3A, RMI1, RMI2	Promotes HR by stimulating 5′ end resection and dissolution of Holliday junction	Doherty et al., 2005; Gravel et al., 2008; Nimonkar et al., 2008; Nimonkar et al., 2011
		RAD51	Inhibits unfavorable HR by melting D-loop	van Brabant et al., 2000; Wu et al., 2001; Bachrati et al., 2006; Bugreev et al., 2007
	SSA	Unknown	Promotes SSA in HEK293 cells, but not in U2OS cells	Sturzenegger et al., 2014
	MMEJ	53BP1	Inhibits MMEJ by interacting with 53BP in G1 cells	Grabarz et al., 2013; Truong et al., 2013
WRN	NHEJ	Ku70, Ku80, DNA-PKcs, XRCC4-LIG4	Promotes NHEJ by stimulating end processing and DNA end ligation	Cooper et al., 2000; Karmakar et al., 2002a; Kusumoto et al., 2008; Sallmyr et al., 2008; Lachapelle et al., 2011
	HR	MRN, EXO1, DNA2, BLM, BRCA1, RPA	Promotes HR by stimulating 5′ end resection by MRN/DNA2 with RPA	Constantinou et al., 2000; Cheng et al., 2004, 2006; Sommers et al., 2005; Lachapelle et al., 2011; Sturzenegger et al., 2014
		RAD51, RAD54	Disrupts potentially deleterious HR intermediates	Otterlei et al., 2006; Opresko et al., 2009
	MMEJ	CtIP, MRE11, PARP1, LIG3	Inhibits end resection by limiting the recruitment of MRE11 and CtIP to DSBs, especially in G1 cells	Sallmyr et al., 2008; Shamanna et al., 2016b
	SSA	DNA2, RAD52	Promotes 5′ end resection and enhances the efficiency of RAD52-mediated strand annealing	Baynton et al., 2003; Sturzenegger et al., 2014
RECQL4	NHEJ	Ku70, Ku80	Promotes NHEJ by interacting with Ku70/80	Shamanna et al., 2014; Lu et al., 2017
	HR	MRE11, NBS1, RAD50, CtIP, BLM, EXO1, DNA2, RAD51	Promotes 5′ end resection by stimulating the recruitment of CtIP, BLM, DNA2, and EXO1 and nuclease activity of MRN	Petkovic et al., 2005; Singh et al., 2012; Lu et al., 2016, 2017
	SSA	Unknown	Inhibits RAD52-mediated SSA	Kohzaki et al., 2020
	MMEJ	Unknown	Promotes MMEJ with an unknown mechanism	Kohzaki et al., 2020
RECQL5	HR	MRN, RAD51	Inhibits exonuclease of MRE11 and RAD51-mediated D-loop formation and displaces RAD51 from ssDNA	Hu et al., 2007; Zheng et al., 2009; Schwendener et al., 2010; Paliwal et al., 2014
	SSA	Unknown	Promotes SSA with an unknown mechanisms in *Drosophila*	Chen et al., 2010

*DSB, double-strand break; BLM, Bloom syndrome protein; WRN, Werner syndrome helicase; NHEJ, non-homologous end joining; HR, homologous recombination; MMEJ, microhomology-mediated end joining; SSA, single-strand annealing.*

### RECQL1

RECQL1, also known as RECQL and RECQ1, is the most abundant member of the five human RecQ helicases (Sharma and Brosh Jr., 2008). It is encoded by the *RECQL1* gene, which is located at chromosome 12p12. RECQL1 consists of 649 amino acid residues, and it possesses the conserved core helicase domain, RecQ Zn^2+^ binding motif, and the RQC domain (Figure 3). RECQL1 can unwind 3′ overhang dsDNA, forked duplexes, 3′ or 5′ flap dsDNA, D-loops, bubble-structured dsDNA, and Holliday junctions, and its helicase activity is stimulated by RPA (Cui et al., 2003; Sharma et al., 2005). RECQL1 plays important roles in DNA repair, restart of stalled replication, and telomere maintenance (Sharma and Brosh Jr., 2007; Popuri et al., 2012a, 2014; Berti et al., 2013; Banerjee et al., 2015; Parvathaneni and Sharma, 2019).

#### RECQL1 and NHEJ

A role for RECQL1 in DSB repair was initially identified *via* the observation that *Recql1* knockout mouse embryonic fibroblasts (MEFs) are highly sensitive to IR and that knockdown of RECQL1 in human cells results in increased cell death to IR and the topoisomerase 1 (TOP1) inhibitor camptothecin (CPT) (Sharma and Brosh Jr., 2007; Sharma et al., 2007). Interestingly, RECQL1 is phosphorylated following exposure to IR and accumulates at IR-damaged chromatin (Sharma and Brosh Jr., 2007), but the IR-induced phosphorylation site(s) and the kinase(s) mediating the RECQL1 phosphorylation have not been identified. A key piece of evidence supporting RECQL1 functions in NHEJ is that it directly interacts with the Ku heterodimer, and does so independently of DNA-PK_*cs*_, and that RECQL1 and Ku70/80 can simultaneously bind linearized plasmid DNA *in vitro* (Parvathaneni et al., 2013). Furthermore, RECQL1 can unwind Ku-bound DNA duplex in a manner that is dependent on intrinsic RECQL1 ATPase activity and RECQL1 modulates end joining in cell-free extracts (Parvathaneni et al., 2013). A reporter-based assay with small interfering RNA (siRNA) library targeting DNA damage response and repair proteins showed that RECQL1 siRNA treatment resulted in a loss of NHEJ efficiency by approximately 25% (Howard et al., 2015). However, exactly how RECQL1 directs end joining and its role in NHEJ *in vivo* are still undefined.

#### RECQL1 and HR

RECQL1 has also been implicated to play a role in HR, as the depletion of RECQL1 by siRNA resulted in increased sister chromatid exchanges in HeLa cells (LeRoy et al., 2005; Sharma and Brosh Jr., 2007). In addition, RECQL1 forms a complex with RAD51 (Sharma and Brosh Jr., 2007) and promotes the processing of Holliday junctions by promoting branch migration (LeRoy et al., 2005; Mazina et al., 2012), indicating that RECQL1 functions in resolving HR intermediates. However, knockdown of RECQL1 in U2OS cells did not significantly reduce HR efficiency, as assessed using a green fluorescent protein (GFP)-based reporter assay (Sharma et al., 2012). RECQL1, similar to RAD51, protects stalled replication forks from MRE11-dependent degradation (Berti et al., 2013; Mason et al., 2019), suggesting that RECQL1 may play an indirect role in the protection and/or repair of one-ended DSBs at replication forks and is not directly required for strand invasion or homology search in HR at two-ended DSBs. Further studies are required to define the mechanism of RECQL1 in the repression of sister chromatid exchanges and its role in HR.

#### RECQL1 and Cancers

The data support that RECQL1 is important for the maintenance of the genome as MEFs from *Recql1* knockout mice show evaluated levels of chromosomal structural aberrations and aneuploidy, and loss of human RECQL1 also causes increased sister chromatid exchanges (LeRoy et al., 2005; Sharma and Brosh Jr., 2007; Sharma et al., 2007). Although no genetic disorders have been identified with mutations in RECQL1, germline mutations in RECQL1 are reported to be linked with an increased risk of breast cancer, suggesting that RECQL1 is a breast cancer susceptibility gene (Cybulski et al., 2015; Kwong et al., 2016; Sun J. et al., 2017; Sun et al., 2015; Tervasmaki et al., 2018). These accumulating clues suggest a tight association between RECQL1 dysfunction and the consequence of genome instability and cancer predisposition (Debnath and Sharma, 2020; Mojumdar, 2020).

### Bloom Syndrome Protein (BLM)

BLM helicase, encoded by the *BLM* gene that is located on chromosome 15q26.1, is a multifunctional protein consisting of 1,417 amino acids. BLM possesses a conserved helicase domain, the RQC domain, and the HRDC domain (Figure 3). BLM can unwind Y-structured dsDNA, bubble-like dsDNA, G-quadruplex, and Holliday junction and also disrupts mobile D-loops (Mohaghegh et al., 2001; Bachrati et al., 2006). The HRDC domain is important for BLM to direct the annealing of DNA strands and the dissolution of double Holliday junctions, but not for unwinding DNA substrates (Cheok et al., 2005; Wu et al., 2005; Gyimesi et al., 2012). An HRDC-dependent conformational change coupled with ATP hydrolysis/DNA translocation cycle promotes BLM to process complex DNA structures (Newman et al., 2015). The N-terminal domain of BLM is unique and conserved in vertebrates, and it is believed to regulate the oligomerization of BLM (Beresten et al., 1999; Janscak et al., 2003; Xu et al., 2012; Shi et al., 2017). BLM participates in different pathways required for DNA metabolism, which it achieves by interacting with many proteins *via* either its N-terminal domain or undefined C-terminal region (Croteau et al., 2014). BLM resolves complex secondary structures such as G-quadruplexes and hairpins during DNA replication and transcription and at telomeres (Schawalder et al., 2003; Johnson et al., 2010; Barefield and Karlseder, 2012; Grierson et al., 2012, 2013; Drosopoulos et al., 2015). BLM can also stabilize stalled replication forks and promote the restart of the stalled forks (Ralf et al., 2006; Pan et al., 2017). BLM functions in multiple DNA repair pathways, with its most-defined role in HR.

#### BLM and HR

Bloom syndrome protein plays a multifaceted role in HR as it is required for the early phase of the pathway (DNA end resection) as well as one of the terminal steps (dissolution of Holliday junctions) (Croteau et al., 2014). Consistent with BLM playing a role at the early and later steps in HR, GFP-tagged BLM accumulates at laser-generated DSBs a few seconds after induction of damage, and it stays at the damage site for hours (Karmakar et al., 2006; Singh et al., 2010). As mentioned above, HR initiates with 5′ DNA end resection by the MRN complex with CtIP and then the 3′ ssDNA is extended by further resection by EXO1 and/or this DNA2/BLM complex. During this step, BLM unwinds dsDNA after binding to 3′ ssDNA to facilitate the endonuclease activity of DNA2, resulting in the generation of an extended 3′ ssDNA (Gravel et al., 2008; Nimonkar et al., 2011). During this process, RPA interacts with BLM and promotes its helicase activity (BroshJr., Li et al., 2000; Doherty et al., 2005; Nimonkar et al., 2011; Soniat et al., 2019; Qin et al., 2020). A recent study reported that CtIP interacts with BLM and stimulates its helicase activity, further promoting DNA2/BLM-mediated extensive resection (Daley et al., 2017). Interestingly, in addition to working with DNA2, another biochemical study found that BLM, but not other RecQ helicases, promotes the nuclease activity of EXO1 on dsDNA (Nimonkar et al., 2008).

Resection-generated 3′ ssDNA is used by RAD51 for strand invasion and exchange with intact sister chromatid, resulting in the formation of a D-loop. BLM can displace the invading strand from the D-loop and thus can disrupt the RAD51–ssDNA filaments, which explains why BLM has also been termed an “anti-recombination” protein, which is a feature observed in Bloom syndrome (BS) patient cells (van Brabant et al., 2000; Wu et al., 2001; Bachrati et al., 2006; Bugreev et al., 2007). The role of BLM as an anti-recombinase is dependent on its helicase activity, but does not require association with DNA topoisomerase IIIα (Patel et al., 2017). BLM stimulates the strand exchange of active ATP-bound RAD51 filaments, but dismantles inactive ADP-bound filaments (Bugreev et al., 2007; Bugreev et al., 2009; Kikuchi et al., 2009), indicating that BLM may not be an actual anti-recombinase but is required to inspect nascent D-loops in order to drive proper HR (Manthei and Keck, 2013; Xue et al., 2019). Another important role of BLM in HR is to process double Holliday junction structures along with topoisomerase IIIα, RMI1, and RMI2, generating only non-crossover recombinant products, which is termed as “dissolution of the Holliday junction” (Wu and Hickson, 2003; Raynard et al., 2006; Wu et al., 2006; Singh et al., 2008; Xu et al., 2008).

#### BLM and Alternative End-Joining Pathways

Although BLM possesses a strong ssDNA annealing activity and is crucial for extensive DNA end resection, its role in the alternative end-joining DSB repair pathways is unclear. Using a GFP-based reporter assay, depletion of BLM by siRNA reduces SSA in HEK293 cells, but not in U2OS cells (Sturzenegger et al., 2014). In contrast, depletion of BLM by short hairpin RNA (shRNA) leads to a significant increase in MMEJ in U2OS cells (Truong et al., 2013). Consistently, another study showed that BLM inhibits MMEJ and long-range CtIP/MRE11-dependent deletions by interacting with 53BP1 in G1 cells, suggesting that BLM plays a role in DSB repair pathway choice (Grabarz et al., 2013).

#### BLM and Diseases

Mutations in BLM lead to a rare autosomal-recessive genetic disorder, Bloom syndrome (BS; OMIM#210900). BS is characterized by growth deficiency, insulin resistance, immune deficiency, photosensitive skin changes, and increased risk for diabetes, as well as high risk of cancer predisposition at a young age (Cunniff et al., 2017; de Renty and Ellis, 2017). Unfortunately, BS patients have a short life span (less than 30 years), and the major cause of death is cancer (Cunniff et al., 2017; de Renty and Ellis, 2017). In addition to BS, heterozygous deleterious mutations in BLM increase the risk of breast cancer (Thompson et al., 2012), prostate cancer (Ledet et al., 2020), and colorectal cancer (Gruber et al., 2002; Cleary et al., 2003; Baris et al., 2007; de Voer et al., 2015). The cells from BS patients display chromosomal instability, which is characterized by elevated rates of chromatid gaps, breaks, sister chromatid exchanges, and quadriradials (Ellis et al., 1995; German et al., 2007). In support of this, an increased frequency of sister chromatid exchanges of >10-fold is used for the standard diagnosis of BS (Chaganti et al., 1974).

### Werner Syndrome Helicase (WRN)

Werner syndrome helicase (WRN) is encoded by the *WRN* gene, which is located at chromosome 8p11-12 (Goto et al., 1992; Yu et al., 1996; Goto et al., 1997). WRN is a 1,432-amino acid-long protein with multiple functional domains, including 3′–5′ exonuclease, conserved helicase domain, RQC domain, and HRDC domain (Croteau et al., 2014; Oshima et al., 2017). The 3′–5′ exonuclease domain in the N-terminal region of WRN is required for multiple DNA repair pathways, and this domain can resolve various DNA substrates, such as fork-shaped duplex, flap-structured dsDNA, D-loops, bubble-structured duplex, Holliday junctions, and G-quadruplexes (Croteau et al., 2014; Oshima et al., 2017). WRN efficiently unwinds duplex DNA with 3′ or 5′ ssDNA tails that are >10 nucleotides long, as well as unwinding duplex DNA duplex with shorter 3′ ssDNA tails (BroshJr., Waheed and Sommers, 2002). The helicase activity of WRN is stimulated by a number of proteins, including RPA, the Ku heterodimer, MRN complex, and the telomere protein TRF2 (Cheng et al., 2004; Sommers et al., 2005; BroshJr., Opresko and Bohr, 2006). Interestingly, a recent study showed that the binding of multiple RPAs super boosts the unwinding activity of WRN so that this helicase can unidirectionally unwind a duplex with a size of >1 kb (Lee et al., 2018). The conserved RQC domain is critical for the substrate-specific DNA binding of WRN to initiate unwinding (Kitano et al., 2010; Tadokoro et al., 2012) and is also required for the ability of WRN to localize at telomere regions, but not at other genomic sites, after oxidative stress (Sun L. et al., 2017). The HRDC domain of WRN plays a role in DNA binding (von Kobbe et al., 2003; Kitano et al., 2007) and is important for the recruitment of WRN protein to DSBs (Lan et al., 2005). In addition, a small region between the RQC and HRDC domains promotes the ability of WRN to execute ssDNA annealing activity and oligomerization (Muftuoglu et al., 2008). The nuclear localization signal is located in the C-terminal region of WRN, and mutations in these sequence result in the translocation of WRN to the nucleolus and account for a significant portion of the mutations that drive the pathogenesis of Werner syndrome (WS) (Matsumoto et al., 1997; Suzuki et al., 2001). Each of these activities of WRN allows it to play a role in multiple DNA-associated metabolisms, including DNA replication, recombination and repair, telomere maintenance, and transcription (Oshima et al., 2017; Shamanna et al., 2017; Mukherjee et al., 2018).

#### Recruitment of WRN to DSB

The WRN plays various roles in NHEJ, HR, MMEJ, and SSA and does so by interacting with key participants in these pathways (Croteau et al., 2014; Shamanna et al., 2017). Cells isolated from WS patients or those with WRN knockdown are sensitive to DSB-inducing agents, including IR, CPT, etoposide, and chromium (Yannone et al., 2001; Imamura et al., 2002; Zecevic et al., 2009; Ammazzalorso et al., 2010). WRN quickly accumulates at laser-induced DSBs and is also retained at the damage sites for many hours (Lan et al., 2005; Singh et al., 2010). The recruitment of WRN to DSBs requires the presence of the HRDC domain, and its recruitment is independent of DSB sensors, such as PARP1, Ku80, DNA-PK_*cs*_, NBS1, and histone H2AX (Lan et al., 2005). In line with WRN’s involvement in multiple DSB repair pathways, the recruitment of this RecQ helicase to DSBs occurs in the G1, S, and G2 phases of the cell cycle (Shamanna et al., 2016b).

#### WRN and NHEJ

The involvement of WRN in NHEJ is supported by the observations that the depletion of WRN results in a reduced *in vitro* ligation of DNA substrates with either blunt or sticky ends, as well as a decrease in NHEJ as monitored by an *in vivo* NHEJ GFP reporter assay (Chen et al., 2003; Shamanna et al., 2016b). WRN physically and functionally interacts with multiple members of the NHEJ machinery, including the Ku heterodimer, DNA-PK_*cs*_, and the XRCC4–LIG4 complex (Cooper et al., 2000; Karmakar et al., 2002a; Kusumoto et al., 2008; Sallmyr et al., 2008; Lachapelle et al., 2011). WRN directly interacts with both subunits of the Ku70/80 heterodimer, which stimulates its exonuclease activity (Cooper et al., 2000; Li and Comai, 2000; Orren et al., 2001). Interestingly, Ku enables WRN to digest DNA containing 8-oxoadenine and 8-oxoguanine modifications, lesions that block the exonuclease activity of WRN in the absence of Ku (Orren et al., 2001). Two putative Ku-binding motifs are located in the N-terminus and C-terminus of WRN, and the interaction between WRN and Ku facilitates the nucleolytic processing of ends (Karmakar et al., 2002b). A recent study reported that these two Ku-binding motifs of WRN function cooperatively to bind the Ku heterodimer and that the N-terminal Ku-binding motif mediates Ku-dependent stimulation of WRN exonuclease activity, promoting DSB repair (Grundy et al., 2016). DNA-PK_*cs*_, phosphorylates WRN at Ser440 and Ser467, and regulates the enzymatic activities of WRN (Yannone et al., 2001; Karmakar et al., 2002a; Kusumoto-Matsuo et al., 2014). The XRCC4-LIG4 complex also interacts with WRN and stimulates its exonuclease activity, but not helicase activity, to generate DNA ends suitable for XRCC4-LIG4-mediated ligation (Kusumoto et al., 2008). Another report indicates that WRN is involved in NHEJ indirectly by upregulating the transcription level of the key NHEJ factor XLF (Liu et al., 2014). Together, the data show that the enzymatic activities of WRN, in particular DNA end processing by the exonuclease activity of WRN, promote NHEJ.

#### WRN and HR

In addition to NHEJ, WRN promotes HR-mediated DSB repair in multiple ways (Saintigny et al., 2002; Chen et al., 2003). WRN participates in DNA end resection during the early stages of HR. WRN interacts with MRE11 and NBS1, which is enhanced by IR, and results in the promotion of WRN helicase activity (Cheng et al., 2004). WRN also physically interacts with DNA2 and promotes extensive DNA end resection in an RPA-dependent matter (Sturzenegger et al., 2014; Pinto et al., 2016). Moreover, during the late S/G2 and M phases of the cell cycle, cyclin-dependent kinase 1 (CDK1) phosphorylates WRN to promote the long-range end resection by DNA2 at replication-associated DSBs, which stimulates HR and the recovery of collapsed replication forks and promotes the maintenance of chromosome stability (Palermo et al., 2016). Specifically, CDK1-dependent phosphorylation of WRN occurs at Ser1133, and this phosphorylation is required for the WRN–MRE11 interaction and promotes MRE11 foci formation at CPT-induced DSBs (Palermo et al., 2016). Moreover, BRCA1 also directly interacts with WRN and stimulates both the helicase and exonuclease activities of WRN, which likely promotes DNA end resection (Cheng et al., 2006). WRN processes intermediates during HR as it promotes the ATP-dependent translocation of Holliday junctions (Constantinou et al., 2000), disrupts mobile D-loop by promoting branch migration, and degrades the invading strand both prior to and after release from the D-loop (Opresko et al., 2009). WRN has also been found to interact with the following HR proteins: RAD51, RAD54, and RAD52 (Baynton et al., 2003; Otterlei et al., 2006; Lachapelle et al., 2011). In addition, the BRCA1/BARD1 complex interacts with WRN *in vivo* and stimulates WRN helicase activity toward forked and Holliday junction substrates (Cheng et al., 2006). RAD52 modulates WRN activity and inhibits its ability to unwind four-way Holliday junctions (Baynton et al., 2003). Collectively, WRN plays important roles in multiple steps during HR.

#### WRN and Alternative End-Joining Pathways

As mentioned above, resected dsDNA with 3′ ssDNA overhangs can be used for HR, MMEJ, and SSA, which is initiated by MRN with the assistance of CtIP (Ronato et al., 2020). Interestingly, WRN actively inhibits DNA end resection by limiting the recruitment of MRE11 and CtIP to DSBs in the G1 phase of the cell cycle (Shamanna et al., 2016b). Accordingly, elevated MMEJ was observed in both WS and WRN-deficient cells, leading to telomere fusions (Shamanna et al., 2016b). In agreement with this finding, limiting MMEJ by depleting CtIP suppresses telomere fusions in WRN-deficient cells (Shamanna et al., 2016b).

WRN interacts with the key SSA player RAD52 and enhances the efficiency of RAD52-mediated strand annealing between non-duplex DNA and homologous sequences contained within a double-stranded plasmid (Baynton et al., 2003). The C-terminal region of WRN may be required for SSA (Muftuoglu et al., 2008). WRN deletion by siRNA causes a 25–50% reduction of SSA-mediated DSB repair in two human cell lines, indicating that WRN promotes SSA (Sturzenegger et al., 2014). However, many questions remain with regard to WRN’s role in SSA, including whether it helps mediate the long resection required for SSA and whether WRN is required for the RAD52-dependent annealing of ssDNA to drive SSA.

#### WRN and Diseases

Mutations in WRN cause the autosomal-recessive disorder Werner syndrome (MIM #277700), which is a segmental progeria (Oshima et al., 2017; Shamanna et al., 2017; Lebel and MonnatJr., 2018). The average life span of WS patients is 54 years, and the major death causes of WS patients are cancer and myocardial infarction (Huang et al., 2006; Goto et al., 2013; Oshima et al., 2017). WS confers a strong predisposition to a diversity of neoplasia, and two thirds of these neoplasia are collectively thyroid neoplasms, malignant melanoma, meningioma, soft tissue sarcomas, leukemia and pre-leukemic conditions of the bone marrow, and primary bone neoplasms (Goto et al., 2013; Lauper et al., 2013). The elevated risk of these neoplasms ranges from 8.9-fold for thyroid epithelial neoplasms to 53.5-fold for melanoma compared to the normal population (Lauper et al., 2013). Meningioma frequently occurs in WS patients (Tsurubuchi et al., 2008; Huang et al., 2018; Pattankar et al., 2020), and this is likely a consequence of a reduced WRN expression due to the elevated methylation of the WRN promoter (Li et al., 2015). In addition, WRN mutations are also associated with other tumors, such as breast cancer (Romanowicz et al., 2017), oral squamous cell carcinoma (Kuribayashi et al., 2019), and colorectal cancer (Lee et al., 2017; Zhunussova et al., 2019; Zimmer et al., 2020). The cells from WS patients display an increased rate of somatic mutations, chromosome losses, deletions, and genomic rearrangements (Subino et al., 1982; Fukuchi et al., 1989; Oshima et al., 2002; Friedrich et al., 2010).

### RECQL4

*RECQL4*, located at chromosome 8q24.3, encodes a 1,208-amino acid protein, which possesses the highly conserved 3′ to 5′ helicase domain, but does not have the typical RQC and HRDC domains (Figure 3). A recent study identified a novel C-terminal domain containing a Zn^2+^-binding site and two distinct winged-helix domains, which are not involved in canonical DNA binding or helicase activity (Kaiser et al., 2017). The N-terminus of RECQL4 contains multiple functional domains, including both nuclear and mitochondrial targeting sequences (Burks et al., 2007; Croteau et al., 2012a). Furthermore, an SLD2-like domain is located in the N-terminus of RECQL4, and it is important for DNA replication (Sangrithi et al., 2005; Xu et al., 2009). Many proteins interact with the N-terminal region of RECQ4 (Croteau et al., 2012b). In addition, functional phosphorylation and acetylation events have also been identified in this region (Dietschy et al., 2009; Lu et al., 2017). Consequently, the N-terminus of RECQL4 is essential for cell viability and for the cellular response to IR (Abe et al., 2011; Lu et al., 2017). The C-terminal region of RECQL4 is not well characterized, but is important for cells to survive IR (Kohzaki et al., 2012). The helicase activity of RECQL4 can unwind forked duplexes, D-loops, and bubble structures, but not duplex DNA or Holliday junctions (Rossi et al., 2010). Interestingly, a recent study identified an unwinding activity of RECQL4 and its yeast homolog Hrq1 on G-quadruplexes (Rogers et al., 2017). RECQL4 has a strong ssDNA annealing activity, which may mask the detection of unwound products (Rossi et al., 2010). The helicase activity is important for the role of RECQL4 in DNA repair and prevention of cellular senescence (Lu et al., 2014, 2016). Overall, RECQL4 is involved in various cellular functions, including DNA replication (Sangrithi et al., 2005), multiple repair pathways (Fan and Luo, 2008; Schurman et al., 2009; Singh et al., 2010; Duan et al., 2020), preservation of the mitochondrial genome (Croteau et al., 2012a), and telomere maintenance (Ghosh et al., 2012).

#### Recruitment of RECQL4 to DSBs

RECQL4-depleted human cells and fibroblasts from Rothmund–Thomson syndrome (RTS) patients are sensitive to IR and other DSB-inducing chemicals, and these cells accumulate unrepaired DSBs, as indicated by elevated γH2AX and 53BP1 foci (Jin et al., 2008; Singh et al., 2010; Kohzaki et al., 2012; Shamanna et al., 2014; Lu et al., 2014, 2016). In *Xenopus* egg extracts, RECQL4 accumulates on chromatin containing *Eco*RI-induced DSBs *in vitro*, which is dependent on RPA, DNA-PK, and ataxia telangiectasia mutated (ATM), but not on DNA replication or RAD51 (Kumata et al., 2007). In human cells, GFP-tagged RECQL4 is recruited immediately to laser−induced DSBs and dissociates from DSBs much more quickly than WRN and BLM, suggesting that RECQL4 only functions at the early time points in DSB repair (Singh et al., 2010; Lu et al., 2016). The unique N−terminus between amino acids 363–492 is the region required for the recruitment of RECQL4 to DSBs (Singh et al., 2010). Moreover, the accumulation of RECQL4 at laser-induced DSBs occurs in both G1 and S/G2 cells (Lu et al., 2017). Inhibition of ATM or knockdown of BLM or WRN does not significantly alter the dynamics of RECQL4 to laser-induced DSBs in human cells (Singh et al., 2010; Lu et al., 2017). The accumulation of RECQL4 at DSBs is stimulated by both phosphorylation at Ser89 and Ser251 by CDK1 and CDK2 and ubiquitination by the DDB1-CUL4A E3 ubiquitin ligase (Lu et al., 2017). However, the ubiquitination sites on RECQL4 by DDB1-CUL4A E3 Ligase have not been identified.

#### RECQL4 and NHEJ

A role for RECQL4 in NHEJ was first indicated when it was found that RECQL4 binds to restriction enzyme-generated DSBs near the Ku70-binding site and that RECQL4 is required for the repair of these DSBs in *Xenopus* extracts (Kumata et al., 2007). Depletion of RECQL4 reduced the end-joining activity on DNA substrates with either cohesive or non-cohesive ends *in vitro* and also decreased the end-joining activity of a GFP reporter plasmid *in vivo*, providing strong evidence that RECQL4 functions in NHEJ in human cells (Shamanna et al., 2014). Further investigations showed that RECQL4 forms a complex with the Ku70/Ku80 heterodimer through its N-terminal domain and stimulates the DNA binding of Ku70/Ku80 to a blunt-ended dsDNA substrate (Shamanna et al., 2014). Interestingly, the interaction of RECQL4 with the Ku complex is enhanced in the G1 phase of the cell cycle compared to that in S/G2 cells, indicating that RECQL4 promotes NHEJ in G1 cells (Lu et al., 2017).

#### RECQL4 and HR

As an important protein in the assembly of DNA replication machinery, RECQL4 is highly expressed during the S phase, the cell cycle phase when HR is the dominant DSB repair pathway, and thus it was predicted that it would also play a role in HR (Sangrithi et al., 2005; Im et al., 2009; Xu et al., 2009; Singh et al., 2012). The initial study suggesting RECQL4 functions in HR reported that it forms a complex with RAD51 following treatment with etoposide (Petkovic et al., 2005). Its role was further elucidated when it was found that the depletion of RECQL4 by siRNAs caused a dramatic loss of HR efficiency, as monitored by a GFP reporter assay, and that RECQL4 is required for DNA end resection (Lu et al., 2016). Specifically, MRE1 regulates the recruitment of RECQL4 to DSBs and RECQL4 promotes the nuclease activity of MRE11 *in vitro*. In addition, RECQL4 also forms a complex with CtIP *via* its N-terminal domain and promotes the recruitment of CtIP to MRN at DSBs. Furthermore, the helicase activity of RECQL4 promotes DNA end processing and HR, and this process is regulated by CDK1/2-mediated phosphorylation (Lu et al., 2017). Specifically, CDK1 and CDK2 phosphorylate RECQL4 on serines 89 and 251, and this facilitates the interaction between MRE11 and RECQL4 as well as RECQL4 recruitment to DSBs (Lu et al., 2017). Interestingly, RECQL4 promotes and coordinates NHEJ and HR in a cell cycle-dependent manner (Lu et al., 2017). RECQL4 preferably interacts with Ku70 to promote NHEJ in G1 when the overall CDK activity is low. During the S/G2 phases, RECQL4 is phosphorylated by CDK1 and CDK2, which promotes the RECQL4–MRN interaction to stimulate HR. These findings indicate a role for RECQL4 in the regulation of DSB repair pathway choice.

#### RECQL4 and Alternative End-Joining Pathways

A study recently discovered a role for RECQL4 in DSB repair pathway choice between MMEJ and SSA (Kohzaki et al., 2020). RECQL4ΔC HCT116 cells (lacking the C-terminal domain) exhibit increased SSA activity and decreased MMEJ activity, and ectopic expression of RECQL4 increased HR and MMEJ but repressed SSA (Kohzaki et al., 2020). Knockdown of RAD52 inhibits SSA in RECQL4ΔC HCT116 cells, but does not influence HR and MMEJ (Kohzaki et al., 2020). The involvement of RECQL4 in the initial end resection by MRN/CtIP may account for the pro-MMEJ role of RECQL4 since a short resected ssDNA is required for MMEJ. However, it is unclear how RECQL4 functions in repressing RAD52-mediated SSA.

#### RECQL4 and Diseases

Mutations in RECQL4 are associated with three rare autosomal-recessive disorders: Rothmund–Thomson syndrome (RTS; OMIM #268400), RAPADILINO (RAPA; OMIM #266280), and Baller–Gerold syndrome (BGS; OMIM #218600) (Siitonen et al., 2009; Larizza et al., 2010, 2013). RTS is accompanied with an increased risk of malignant tumor osteosarcoma in childhood (Wang et al., 2003; Larizza et al., 2010; Lu et al., 2020). RAPADILINO patients develop lymphoma and osteosarcoma (Siitonen et al., 2009), while a BGS patient was reported with lymphoma (Debeljak et al., 2009). In addition, germline mutations of RECQL4 have also been identified to associate with several cancers, including prostate cancer (Paulo et al., 2018), colorectal cancer (Zhunussova et al., 2019), choroid plexus papilloma (Taher et al., 2019), and melanoma (Bodelon et al., 2012; Aoude et al., 2020). Chromosome aberrations, mainly mosaic trisomies and isochromosomes, frequently occur in RTS patient cells, leading to the cancer predisposition of these patients (Larizza et al., 2006). In line with this finding in human RTS cells, defective sister chromatid cohesion, aneuploidy, and cancer predisposition were observed in the cells from an RTS mouse model (Mann et al., 2005). Therefore, RECQL4 is crucial to maintain the integrity of the genome.

### RECQL5

RECQL5, encoded by the *RECQL5* gene at chromosome 17q25, exists in three isoforms generated by alternative splicing: RECQL5α, RECQL5β, and RECQL5γ. RECQL5α and RECQL5γ consist of 410 and 435 amino acids, respectively, while RECQL5β contains 991 amino acids (Figure 3) (Sekelsky et al., 1999; Shimamoto et al., 2000). All three isoforms have the core helicase domain, while only RECQL5β has helicase activity *in vitro* (Shimamoto et al., 2000). Additionally, RECQL5β (referred to as RECQL5 in the following text) has a Zn^2+^-binding motif and a unique C-terminal region harboring multiple specific protein interaction domains: a RAD51-binding domain, internal RNAPII-interacting (IRI) domain, Set2-Rpb1-interacting (SRI) domain, RNA polymerase I (RNAPI)-binding domain, and a proliferating cell nuclear antigen (PCNA)-interacting protein (PIP) motif (Andrs et al., 2020). The Zn^2+^-binding motif is essential for helicase activity and DNA binding (Ren et al., 2008). RECQL5 has intrinsic ssDNA strand annealing activity, which is inhibited by ATP (Garcia et al., 2004; Ren et al., 2008). However, a recent study showed that RECQL5 possesses a stronger annealing activity on long or small duplexed substrates compared to the other RecQ helicases, which is not inhibited by the presence of ATP (Khadka et al., 2016). RECQL5 efficiently catalyzes the annealing of RNA to DNA *in vitro* in the presence or absence of ATP (Khadka et al., 2016). However, the cellular function of this activity for RECQL5 is yet to be delineated.

#### Recruitment of RECQL5 to DSBs

RECQL5-depleted cells accumulate persistent IR-induced 53BP1 foci, implicating a role for RECQL5 in DSB repair (Popuri et al., 2012b). RECQL5 is recruited quickly to laser-induced DSBs and remains for a shorter duration than BLM and WRN, but persists longer than RECQL4 (Zheng et al., 2009; Popuri et al., 2012b). Both the helicase and KIX domains are required for DNA damage recognition and the stable association of RECQL5 to DSB sites (Popuri et al., 2012b). The recruitment of RECQL5 requires MRE11, but not the exonuclease activity of MRE11, and its recruitment to DSB is independent of RNA polymerase II, BLM, WRN, ATM, MDC1, and CtIP (Zheng et al., 2009; Popuri et al., 2012b). RECQL5 interacts with Poly(ADP-ribose) (PAR) and Poly(ADP-ribose) polymerase 1 (PARP1), and PARylation by PARP1 stimulates the recruitment of RECQL5-GFP to laser-induced DSBs (Khadka et al., 2015).

#### RECQL5 and HR

Deletion of RECQL5 increases HR in MEFs (Hu et al., 2007). Consistent with this, it was found that RECQL5 binds to RAD51, inhibits RAD51-mediated D-loop formation, and displaces RAD51 from ssDNA in assistance with ATP hydrolysis and RPA (Hu et al., 2007; Schwendener et al., 2010). Another study reported that RECQL5 counteracts the inhibitory effect of RAD51 on RAD52-mediated DNA annealing with its ATPase and RAD51-binding activity (Paliwal et al., 2014). Furthermore, RECQL5 deficiency causes an increased occupancy of RAD51 at DSBs and evaluated sister chromatid exchange when the Holliday junction dissolution pathway is inactivated or a high load of DNA damage is generated in the cell (Paliwal et al., 2014). Together, these findings suggest that RECQL5 functions during the postsynaptic phase of synthesis-dependent strand annealing to prevent the formation of aberrant RAD51 filaments on the extended invading strand and inappropriate HR events (Hu et al., 2007; Schwendener et al., 2010; Paliwal et al., 2014). RECQL5 forms a constitutive complex with MRN and specifically inhibits the exonuclease activity of MRE11 (Zheng et al., 2009), but it is unclear whether this activity plays a role in the ability of RECQL5 to suppress HR. Interestingly, it was reported that RECQL5 promotes HR-mediated repair with non-crossover products in a direct repeat GFP (DR-GFP) reporter assay in both U2OS and HEK293 cells (Paliwal et al., 2014; Khadka et al., 2015). RECQL5 deficiency in *Drosophila* causes sensitivity to IR and DSBs induced by the I-SceI endonuclease and impairs SSA-mediated DSB repair (Chen et al., 2010), but it is unknown whether RECQL5 modulates SSA in mammalian cells.

#### RECQL5 and Cancers

The downregulation of RECQ5 leads to a transcription-dependent chromosome fragmentation during S phase of the cell cycle as well as the accumulation of chromosomal rearrangements with the breakpoints located in genes and common fragile sites, indicating the importance of this helicase in maintaining genome integrity (Li et al., 2011; Saponaro et al., 2014). Although defects in RECQL5 have not been associated with human genetic disorders, mutations in RECQL5 have been associated with tumorigenesis, including breast cancer (He et al., 2014), osteosarcoma (Zhi et al., 2014; Dong et al., 2015), NUT midline carcinoma (Stirnweiss et al., 2017), head and neck cancer (Das et al., 2018), and hereditary diffuse gastric cancer (Fewings et al., 2018).

## Perspective Remarks

A significant number of clues have uncovered the roles that the RecQ helicases play in DSB repair and the maintenance of genome stability. Furthermore, the evidence has started to reveal how defects in the RecQ helicases drive specific genetic disorders and carcinogenesis. However, many questions still remain, including: (1) a clear role for each RecQ helicase’s enzymatic activity in DSB repair is yet to be demonstrated; (2) it is still unclear how BLM, WRN, and RECQL4 cooperate in 5′ DNA end resection during HR; (3) RECQL4 interacts with Ku70/80 heterodimers and is required for NHEJ, but its role in this pathway is unknown; (4) the role of RecQ helicases in MMEJ and SSA are also limited; (5) does RECQL5 play a role in RNA-mediated DSB repair; (6) do the RecQ helicases play a direct or passive role in DSB repair pathway choice; (7) what role does posttranslational modifications play in regulating the RecQ helicases; and (8) can RecQ helicases be targeted for specific cancer therapies. Among these questions, investigating the posttranslational modifications of RecQ helicases may shed light on many unknown mechanisms, including the enzymatic roles of RecQ helicases in specific steps of DSB repair pathways, coordination between the RecQ helicases during specific repair processes, and the role(s) of RecQ helicases in DSB repair pathway choice. Thanks to the great advance of mass spectrometry, hundreds of posttranslational modifications, including phosphorylation, acetylation, ubiquitination, etc., have been identified on RecQ helicases^1^ (Hornbeck et al., 2012, 2015, 2019). However, the biological functions of these modifications are yet to be elucidated. Hence, it would be promising to investigate posttranslational modifications of RecQ helicases with the combination of spatial and temporal factors as well as stresses. Recently, WRN was identified as a synthetic lethal vulnerability in cancers with microsatellite instability (Chan et al., 2019; van Wietmarschen et al., 2020). Interestingly, CPT treatment leads to the degradation of WRN (Shamanna et al., 2016a; Li et al., 2020), suggesting that cancers with microsatellite instability may be a candidate target for CPT treatment to drive WRN-specific vulnerable tumors. These findings together demonstrate WRN as an important genome guardian to prevent diseases and as a potential drug target. Collectively, these and other unanswered questions drive us and others to understand the role of each RecQ helicase in DSB repair, how defects drive human disorders, and how these enzymes may be targets for therapy.

## Author Contributions

HL and AD conceived the concept and wrote the manuscript together. Both authors contributed to the article and approved the submitted version.

## Conflict of Interest

The authors declare that the research was conducted in the absence of any commercial or financial relationships that could be construed as a potential conflict of interest.

## Footnotes

1 www.phosphosite.org
